# Butyl and isobutyl ortho-coumaric acid derivatives reduce self-renewal and MGMT expression in GBM cells with stem cell-like characteristics *in vitro*

**DOI:** 10.17305/bb.2026.13514

**Published:** 2026-05-29

**Authors:** Luis Javier Reséndiz-Castillo, Yanet Karina Gutiérrez-Mercado, Juan Carlos Mateos-Díaz, Ahtziri Socorro Carranza-Aranda, Edwin Estefan Reza-Zaldivar, Mercedes Azucena Hernández-Sapiéns, Alejandro Arturo Canales-Aguirre

**Affiliations:** 1 Preclinical Evaluation Unit, Center for Research and Assistance in Technology and Design of the State of Jalisco, Guadalajara, Jalisco, Mexico; 2 Biotechnological Laboratory for Research and Diagnosis, Department of Clinics, University of Guadalajara, Campus Los Altos, Tepatitlán de Morelos, Jalisco, Mexico; 3 Industrial Biotechnology Unit, Center for Research and Assistance in Technology and Design of the State of Jalisco, Zapopan, Jalisco, Mexico; 4 Department of Philosophical, Methodological and Instrumental Disciplines, University of Guadalajara, Health Sciences Campus, Guadalajara, Jalisco, Mexico; 5 School of Medicine and Health Sciences, Tecnológico de Monterrey, Monterrey, Mexico; 6 School of Engineering and Sciences, Tecnológico de Monterrey, Monterrey Campus, Monterrey, Nuevo León, Mexico

**Keywords:** Glioma, glioblastoma, coumaric, brain tumor, cancer, stem cell, phenolic compound

## Abstract

Ortho-coumaric acid (o-CA) derivatives, specifically butyl, isobutyl, propyl, ethyl, and methyl-o-coumarate, have emerged as potential therapeutic agents for glioblastoma multiforme (GBM). This study investigates their effects on drug resistance, proliferation, migration, maturation, cell cycle regulation, and autophagy in stem-like glioblastoma cells (SLGCs). We evaluated the biological activity of o-CA derivatives using an *in vitro* GBM cell model by assessing spheroid and colony formation, cell migration, autophagy marker induction, DNA damage repair protein expression, cell cycle alterations, and the expression of stemness and differentiation markers. Treatment with o-CA derivatives, particularly butyl and isobutyl, resulted in a decreased self-renewal capacity of SLGCs, as indicated by reduced spheroid and colony formation compared to temozolomide (TMZ). Additionally, butyl o-CA impaired cell migration. Cell cycle analysis revealed an increased proportion of cells in the G0/G1 phase following treatment with butyl and isobutyl o-CA, accompanied by a reduction in the S and G2/M phases. Furthermore, we observed increased expression of the autophagy marker microtubule-associated protein 1 light-chain 3B (LC3-B) in cells treated with propyl and butyl o-CA. The expression of the drug resistance marker O6-methylguanine DNA methyltransferase (MGMT) was significantly reduced, particularly in cells treated with Butyl o-CA. Moreover, butyl o-CA-treated cells demonstrated lower expression levels of the stemness markers CD133 and Nestin, in conjunction with elevated expression of the differentiation markers GFAP and NeuN. Overall, these findings suggest that o-CA derivatives, especially butyl and Isobutyl o-CA, may effectively modulate stem-like characteristics in an *in vitro* GBM model.

## Introduction

Glioblastoma multiforme (GBM) is the most common and aggressive primary brain tumor affecting the central nervous system. It constitutes 50% of all gliomas, 52% of parenchymal brain tumors, and 20% of intracranial tumors, with an incidence rate of 3 cases per 100,000 inhabitants [1]. Despite notable advancements in treatment strategies, patient prognosis remains poor, with a median survival time of approximately 12–18 months [2]. Resistance to standard therapies and a high recurrence rate pose significant challenges in the clinical management of GBM [3].

**Figure 1.**
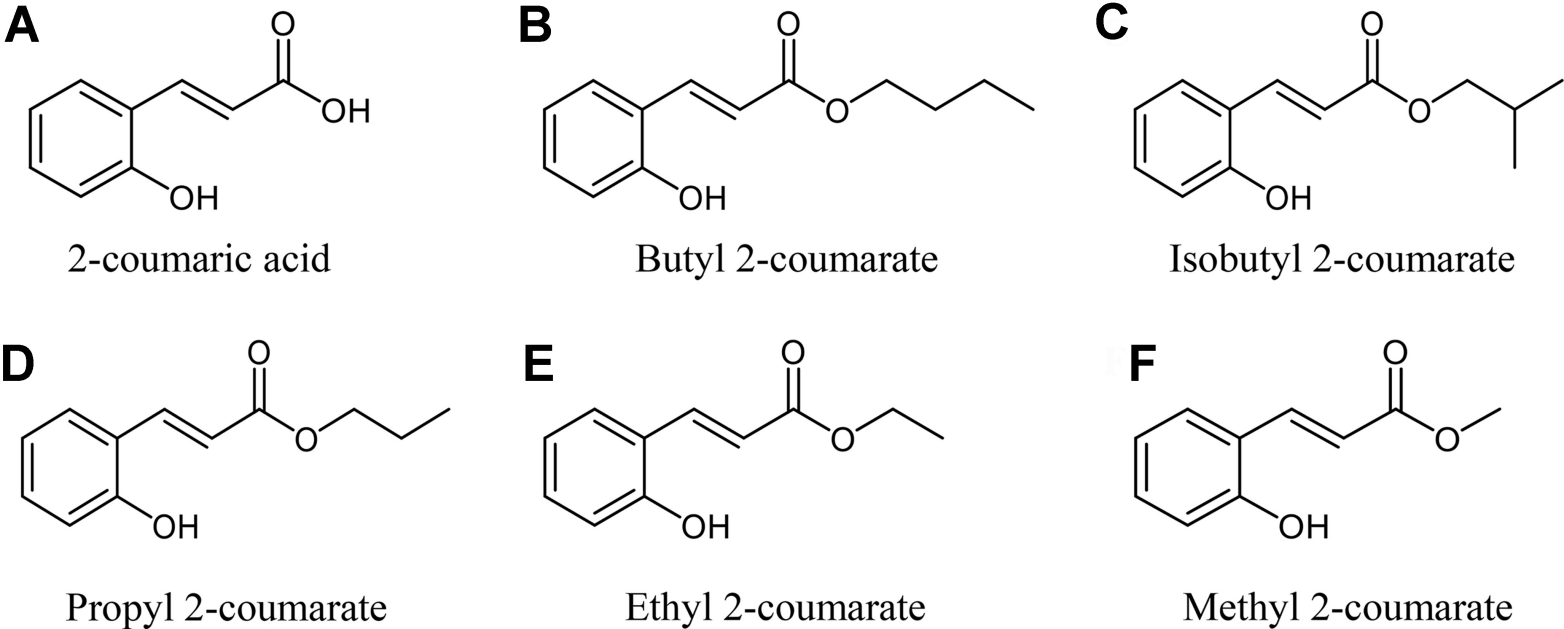
**Chemical structures of o-CA and o-CA ester derivatives used in this study.** Chemical structures are shown for (A) o-CA and its ester derivatives: (B) Butyl-o-CA, (C) Isobutyl-o-CA, (D) Propyl-o-CA, (E) Ethyl-o-CA, and (F) Methyl-o-CA. Structures were drawn using ChemDraw. Abbreviation: O-CA: Ortho-coumaric acid.

The heterogeneity of the tumor microenvironment (TME) in GBM, along with the presence of stem-like glioblastoma cells (SLGCs), has been identified as a critical factor in tumor progression and therapeutic resistance [4]. SLGCs within the TME enhance the malignancy of the tumor by increasing self-renewal capabilities while concurrently reducing the potential for differentiation among various neural populations. This dynamic contributes to tumor recurrence and resistance to conventional multimodal therapies, including surgical resection, radiotherapy, and chemotherapy with Temozolomide (TMZ) [5, 6]. Despite these aggressive treatment approaches, their overall effectiveness remains limited, as only 2% to 3% of patients survive beyond two years post-treatment [2].

In this context, the utilization of biomarkers in GBM has facilitated a deeper understanding of the molecular features driving tumor malignancy. Several proteins significantly enhance SLGC properties by promoting cell migration and proliferation and inhibiting GBM cell differentiation. Notable biomarkers include the overexpression of O6-methylguanine DNA methyltransferase (MGMT), which contributes to resistance against alkylating agents; mutations in isocitrate dehydrogenase 1 (IDH1); overexpression of the epidermal growth factor receptor (EGFR); and reduced expression of microtubule-associated protein 1 light chain 3B (LC3-B), all associated with increased migration, proliferation, and drug resistance [7, 8]. Additionally, biomarkers such as CD133 and Nestin indicate the capacity of SLGCs to maintain an undifferentiated cellular state, rendering them more aggressive and challenging to treat. Conversely, proteins like neuronal nuclei (NeuN) and glial fibrillary acidic protein (GFAP) reflect the maturation of neuronal and astrocytic cells, with their expression decreased in differentiated tumor stem cells [9, 10]. These biomarkers are valuable tools for identifying tumor cell subpopulations and developing targeted therapies for GBM.

Consequently, the search for more effective therapies centered on natural or naturally derived compounds, such as phenolics with anticancer potential, is of paramount importance [11]. These bioactive compounds have demonstrated anticancer activity [12], particularly ortho-coumaric acid (o-CA), which has garnered interest due to its antioxidant and anticancer properties, including the activation of tumor suppressor genes such as p53, Bax, caspase-8, and caspase-3 [11, 13]. In our previous research, we established that o-CA and its ester derivatives exert cytotoxic and pro-apoptotic effects on the U138-MG glioblastoma cell line, evidenced by reduced cell viability, induction of apoptosis, activation of caspase-3, annexin V positivity, and DNA fragmentation, with Butyl and Isobutyl o-CA exhibiting particularly promising anticancer activity compared to TMZ [14].

Despite these encouraging findings, the broader therapeutic potential of o-CA derivatives in glioblastoma—particularly their effects on drug resistance mechanisms and their interaction with SLGCs—has yet to be comprehensively investigated. Our earlier work primarily focused on acute cytotoxicity in the U138-MG model, which is commonly employed for apoptosis and viability studies, while the U87-MG cell line has been extensively used to explore glioblastoma behaviors related to tumorigenic potential, stemness, and differentiation, including the characterization of subpopulations with cancer stem-like properties [15]. Building on our previous observations, this study extends the evaluation of o-CA derivatives to the U87-MG glioblastoma model, concentrating on tumorigenic properties beyond cytotoxicity. Specifically, we assess tumor spheroid-forming capacity, clonogenic survival, cell cycle arrest, and modulation of molecular markers associated with stemness, differentiation, DNA repair, and autophagy. Through this approach, we aim to provide further insights into the potential of o-CA derivatives as candidate adjuvant agents relevant for future multimodal GBM treatment strategies.

## Materials and methods

### o-CA derivatives

The o-CA derivatives utilized in this study included Butyl-o-coumarate (Butyl-o-CA), Isobutyl-o-coumarate (Isobutyl-o-CA), Propyl-o-coumarate (Propyl-o-CA), Ethyl-o-coumarate (Ethyl-o-CA), and Methyl-o-coumarate (Methyl-o-CA) (Figure 1). These derivatives were synthesized and purified as previously reported by Gutiérrez Mercado et al. (2022). TMZ (Sigma-Aldrich, catalog No. PHR1437-1G) served as a control treatment, while dimethyl sulfoxide (DMSO) (Sigma-Aldrich, catalog No. D2650) acted as a vehicle.

### Cell culture

The GBM U87-MG cell line from the American Type Culture Collection (ATCC; Rockville, USA) was cultured in Dulbecco’s modified Eagle’s minimal essential medium (DMEM, Sigma-Aldrich, D6429), supplemented with 10% inactivated fetal bovine serum (FBS) (Corning™ , 35-016-CV), 1% pyruvate, 1% non-essential amino acids, and 1% antibiotic-antimycotic (Gibco^®^ , 15240062). The cells were maintained at 37 ^∘^C in a humidified atmosphere with 5% CO2. Routine testing for mycoplasma contamination was conducted using polymerase chain reaction (PCR)-based assays, confirming that the cells were mycoplasma-free. Cell line authentication was provided by the supplier (ATCC). For all experiments, untreated cells served as a control condition. Cultures were then treated with o-CA derivatives at concentrations previously established as biologically active in U87-MG cells, or with TMZ and DMSO as a vehicle group (Table 1). These concentrations were based on functional data presented at a scientific conference [16] and supported by a granted patent [17]; however, these data have not yet been published.

**Table 1:** Concentrations of o-CA derivatives, TMZ, and vehicle

**o-CA Derivatives**	**Concentration**
Butyl-o-coumarate	200 µM
Isobutyl-o-coumarate	200 µM
Propyl-o-coumarate	300 µM
Ethyl-o-coumarate	300 µM
Methyl-o-coumarate	300 µM
TMZ	300 µM
Vehicle (DMSO)	0.5 %

Abbreviations: O-CA: Ortho-coumaric acid; TMZ: Temozolomide; DMSO: Dimethyl sulfoxide.

### Tumor sphere-forming assay

The assay was conducted according to protocols established in [18–20] with minor modifications. Cells were initially cultured in a monolayer under standard conditions until primary spheroids formed [21]. Spheroids were measured, and those larger than 100 µm were considered primary spheroids. These primary spheroids were collected and mechanically dissociated into single-cell suspensions. The resultant tumor-sphere-forming cells were seeded at a density of 100 cells per well in 96-well plates to generate secondary spheroids and treated for 24 h with o-CA derivatives and TMZ at the aforementioned concentrations. Following the treatment period, the medium was replaced with fresh standard medium, and cultures were maintained for 14 days, with medium replacement every 48 h. At the end of the culture period, the number, size, and shape of secondary spheroids were evaluated. Spheroid shape and size were assessed using Leica Application Suite (LAS) software, and the number of secondary spheroids formed per well was used as the primary endpoint for analysis [22].

### Clonogenic colony formation assay

To assess the clonogenic capacity of cells in monolayer culture, 100 cells per well were seeded in 6-well plates. Cells were treated for 24 h with o-CA derivatives and TMZ. Following treatment, the medium was replaced with fresh medium, and the cultures were maintained for 14 days, with medium replacement every 48 h. At the end of this period, the cells were fixed with 4% paraformaldehyde and stained with a 0.5% crystal violet solution. Colony growth and morphology were examined using a stereoscope (Leica EZ4). The total number of colonies formed was quantified using the “OpenCFU” software, considering only colonies with 50 or more cells.

### Cell migration assay

The effect of o-coumaric derivatives on the migration ability of U87-MG cells was evaluated in a 12-well plate seeded with 50,000 cells per well until reaching 80% confluency in a monolayer. Subsequently, two linear wounds were created in the cell monolayer using a 200 µL pipette tip (600 µm) along the diameter of the well. Floating cells were washed away with phosphate-buffered saline (PBS), and cells were incubated for 24 h with o-CA derivatives and TMZ. Following this incubation period, wound healing was evaluated. Images of the wounds were captured at 0 h and after 24 h of treatment using an inverted phase-contrast microscope (Olympus IX71) (DM-IL-LED-FLUO) and analyzed using LAS software, version 3.6.0.20104. The analysis measured the cell-free distance between both boundaries of the wound in the monolayer. This distance was calculated using ImageJ software.

### Cell cycle arrest by flow cytometry

The cell cycle arrest induced by o-CA derivatives was analyzed in 6-well plates, each seeded with 20,000 cells until reaching 80% confluence in a monolayer. The culture medium was replaced with DMEM lacking FBS and supplemented with the respective treatments. Following this, the cells were incubated for 24 h with o-CA derivatives and TMZ. Subsequently, the cells were collected and stained with propidium iodide buffer (0.05 mg/mL) while being agitated for 1 minute. Afterward, the cells were incubated for 20 min at 4 ^∘^C in the dark.

Flow cytometric analysis was performed using the BD Accuri C6 Plus cytometer, with data acquisition of 10,000 events per sample. Prior to cell cycle analysis, cellular debris was excluded based on a forward scatter (FSC) threshold of 80,000. Doublets and cell aggregates were eliminated using FSC-area (FSC-A) vs FSC-height (FSC-H) gating. The distribution of cell cycle phases (G0/G1, S, and G2/M) was determined using FlowJo software (version 10.6).

### Immunofluorescence of drug resistance and autophagy-associated proteins

To assess drug resistance in U87-MG cells, the cells were seeded at a density of 5,000 cells per well in an 8-well chamber slide for 24 h. Following this, the cells were treated with o-CA derivatives and TMZ for 72 h. The medium was removed and the cells were washed three times with PBS. The cells were then fixed with 4% paraformaldehyde for 15 min at room temperature (RT), permeabilized with 0.5% Triton X-100 in PBS for 1 hour at RT, and blocked with 10% normal goat serum for 1 h at RT. Primary antibodies against MGMT (1:1000; Santa Cruz sc-166528) and LC3-B (1:500; Santa Cruz sc-376404) were incubated overnight at 4 ^∘^C. Alexa 488 anti-mouse secondary antibody (1:500; Vector Laboratories) was applied and incubated for 2 h at RT. Images were captured using a Leica upright microscope (DM4 DF7000T) with a 10× objective and analyzed using the LAS X software (version 3.6.0.20104). Quantification of fluorescence was expressed in Normalized Fluorescent Units (NFU). Fluorescence intensity was quantified using ImageJ software (National Institutes of Health, Bethesda, MD). For each experimental condition, three randomly selected fields were analyzed, each containing at least three cells. A region of interest (ROI) was manually drawn over the specific fluorescent signal of the marker of interest, while a second ROI of comparable size was placed in an adjacent cell-free area to measure background fluorescence. The same background-correction procedure was applied to the 4′,6-diamidino-2-phenylindole (DAPI) channel. Background-corrected nuclear fluorescence was used for normalization. NFU was calculated by dividing the background-corrected fluorescence intensity of the marker by the background-corrected DAPI fluorescence for each field. Fluorescence quantification was conducted following established ImageJ-based protocols for background correction and normalization [23]. No primary antibody negative controls were included; background fluorescence was assessed and corrected using adjacent cell-free regions for each field.

### Immunofluorescence of differentiation and stem cell markers

The expression of CD133 (eBioscience™ 14133180), Nestin (Santa Cruz sc-23927), NeuN (Abcam Ab18102), and GFAP (Santa Cruz sc-33673) was evaluated using the immunofluorescence protocol previously described. All primary antibodies were used at a 1:500 dilution and incubated overnight at 4 ^∘^C. Secondary antibodies, Alexa 488 anti-mouse and Alexa 594 anti-rabbit, were used at a 1:500 dilution and incubated for 2 h at RT. Images were acquired using a Leica confocal microscope (TCS SPE) and analyzed in the LAS X software (version 3.1.1.15751). For each experimental condition, three randomly selected fields were analyzed, each containing at least three cells. ROIs were manually drawn over the specific fluorescent signal of the markers of interest, while additional ROIs of comparable size were placed in adjacent cell-free areas to measure background fluorescence. The same background-correction procedure was applied to the DAPI channel. Background-corrected nuclear fluorescence was utilized for normalization. NFU was calculated by dividing the background-corrected fluorescence intensity of the marker by the background-corrected DAPI fluorescence for each field. No primary antibody negative controls were included; background fluorescence was assessed and corrected using adjacent cell-free regions for each field.

### Ethical statement

This study did not involve human participants or animal experimentation. All experiments were conducted using established commercially available cell lines; therefore, no institutional review board or ethics committee approval was required.

### Statistical analysis

Statistical analyses were chosen based on data type (parametric or nonparametric; continuous or discrete). Normality was assessed using the Shapiro–Wilk test, and homogeneity of variances was evaluated using Levene’s test. Q–Q plots and histograms were further inspected for each experiment to ensure the appropriate selection of statistical tests.

Colony and spheroid counts were analyzed using count regression models (Poisson regression). Model assumptions were evaluated through inspection of residuals and assessment of potential overdispersion. Post hoc comparisons were conducted using Dunnett’s adjustment, which inherently accounts for multiple testing within each endpoint. Results are reported as model-predicted counts (estimated marginal means) with 95% confidence intervals (CIs).

Wound closure data were analyzed using beta regression models that included treatment, time, and their interaction. Dunnett-adjusted post hoc comparisons were performed to compare each treatment with the untreated control and TMZ groups at 24 h, based on estimated marginal means probabilities with 95% CIs.

Cell cycle phase distribution was analyzed using a multinomial regression model, with treatment as the main predictor for each phase. Estimated marginal probabilities and 95% CIs were obtained for each phase. Dunnett-adjusted post hoc comparisons were conducted to compare each treatment with the control and TMZ groups.

MGMT, LC3-B, CD133, Nestin, NeuN, and GFAP expression levels were quantified as NFU. Given the wide dynamic range of fluorescence-based measurements, data for all markers were log10-transformed prior to statistical analysis. Differences in MGMT and LC3-B expressions among treatments were assessed using one-way analysis of variance (ANOVA) followed by Dunnett’s multiple comparisons test against the untreated control and TMZ groups. Expression analyses of CD133, Nestin, NeuN, and GFAP were conducted using two-way ANOVA followed by Dunnett’s multiple comparisons test under the same comparison scheme. Data are presented as log-transformed NFU means with 95% CIs.

Each assay was performed using at least three independent biological replicates, with technical replicates included when applicable. No additional false discovery rate (FDR) correction was applied, as Dunnett’s adjustment was utilized for all post hoc comparisons within each analysis. Statistical analyses were performed using RStudio, and graphical representations were generated using GraphPad Prism version 10.

Statistical significance was defined as *P* < 0.05 (*), P < 0.01 (**), P < 0.001 (***), and *P* < 0.0001 (#).

## Results

### Effect of o-coumaric derivatives on spheroid formation

To evaluate the impact of o-CA derivatives on spheroid formation, a spheroid formation assay was conducted using a serum-derived tumor spheroid model, which is commonly utilized in glioblastoma research [21, 24, 25]. The results demonstrated that treatment with o-CA derivatives significantly impaired spheroid formation, resulting in a decrease in the number of spheroids and alterations in their morphology, including irregular edges and reduced size compared to untreated control cells.

**Figure 2.**
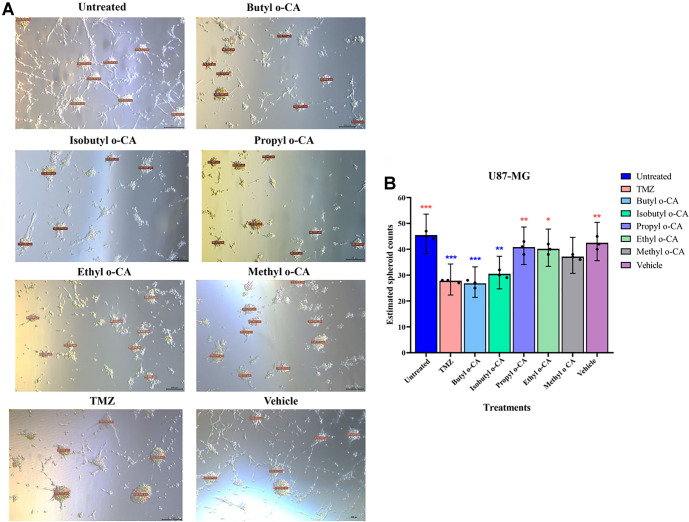
**Effect of o-CA derivatives on inhibition of spheroid formation in U87-MG cells**. (A) Representative images of spheroids treated with o-CA, TMZ, or vehicle for 24 h and subsequently cultured for 14 days. Scale bar: 200 µm. (B) Quantification of totalspheroids formed following treatment with o-CA derivatives, TMZ, or vehicle. Data are presented as estimated marginal means (model-predicted spheroid counts) derived from a Poisson regression model, with 95% CIs. Dunnett-adjusted post hoc comparisons were conducted against the untreated control and TMZ; *P* < 0.05; *P < 0.01; ***P < 0.001; #*P* < 0.0001. Blue symbols indicate statistical significance compared to the control, while red symbols denote significance relative to TMZ. Abbreviations: o-CA: Ortho-coumaric acid; U87-MG: Human glioblastoma cell line; TMZ: Temozolomide; CIs: Confidence intervals.

Treatment with o-CA derivatives markedly affected the spheroid formation capacity in U87-MG cells. The estimated spheroid counts showed a significant reduction following treatment with Butyl o-CA (26.7), Isobutyl o-CA (30.3), and TMZ (27.7) compared to the control (45.3). In contrast, Ethyl o-CA (40.0), Propyl o-CA (40.7), Methyl o-CA (37.0), and the vehicle control (42.3) exhibited a lesser tendency to reduce the number of spheroids (Figure 2; Table S1).

Additionally, compared to the effect of TMZ, the molecules Propyl o-CA, Ethyl o-CA, and Methyl o-CA significantly promoted an increase in spheroid formation. Thus, these findings indicate that only certain o-CA derivatives reduce spheroid formation in U87-MG cells (Figure 2; Table S1).

### Effect of o-coumaric derivatives on colony formation

**Figure 3.**
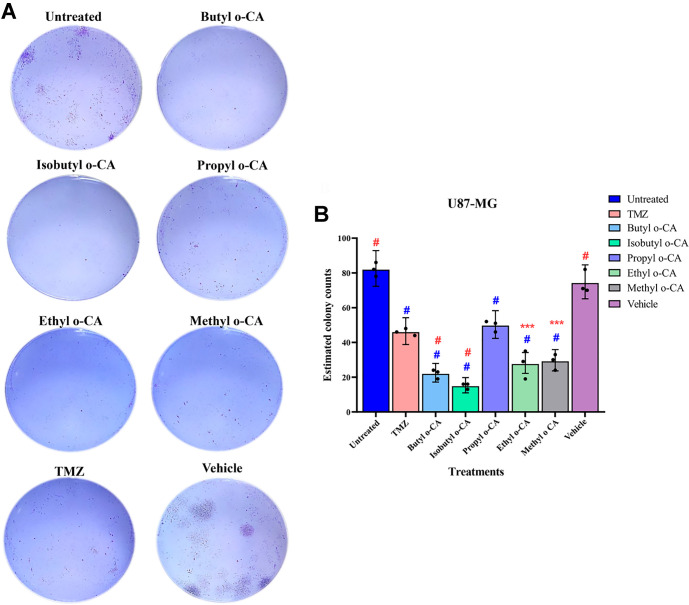
**Effect of o-CA derivatives on the inhibition of colony formation in U87-MG cells.** (A) Representative images of U87-MG cells treated with o-CA derivatives, TMZ, and vehicle for 24 h, followed by a 14-day culture period. (B) Quantification of the total number of colonies formed following treatment with o-CA derivatives, TMZ, or vehicle. Data are presented as estimated marginal means (model-predicted colony counts) derived from Poisson regression analysis, including 95% confidence intervals. Dunnett-adjusted post hoc comparisons were performed against untreated controls and TMZ; *P* < 0.05; *P < 0.01; ***P < 0.001; #*P* < 0.0001. Blue symbols indicate statistical significance compared to control, while red symbols indicate significance compared to TMZ. Abbreviations: o-CA: Ortho-coumaric acid; U87-MG: Human glioblastoma cell line; TMZ: Temozolomide.

The clonogenic effect and self-renewal capacity of U87-MG cells were significantly diminished by selected o-CA derivatives. The results indicated a substantial reduction in estimated colony counts following treatment with Isobutyl o-CA (14.8), followed by Butyl o-CA (21.9), Ethyl o-CA (27.6), Methyl o-CA (29.1), TMZ (45.9), and Propyl o-CA (49.7) compared to untreated (81.9) and vehicle (74.2) control cells. TMZ treatment also significantly reduced colony counts, albeit to a lesser extent than most o-CA derivatives, except for Propyl o-CA and the untreated and vehicle control cells (Figure 3; Table S2).

### Decreased migration capacity

To evaluate the impact of o-CA derivatives on the migratory capacity of glioblastoma cells, a wound-healing assay was conducted. Wound closure dynamics were analyzed using a beta regression model to accommodate the proportional nature of the data. Overall, both treatment and time significantly influenced wound closure behavior.

**Figure 4.**
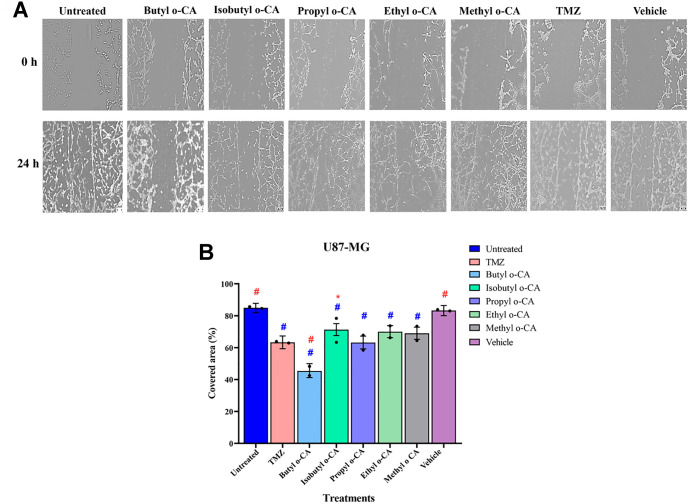
**Effect of o-CA derivatives on the inhibition of U87-MG cell migration.** (A) Representative images from wound healing assays taken at 0 h (immediately after wound creation) and 24 h post-treatment with o-CA derivatives. (B) Quantification of wound closure after 24 h of treatment with o-CA derivatives. Data are expressed as estimated marginal probabilities (%) derived from a beta regression model, accompanied by 95% confidence intervals. Dunnett-adjusted comparisons against the control and TMZ are presented; *P* < 0.05; *P < 0.01; ***P < 0.001; #*P* < 0.0001. Blue symbols indicate a statistically significant difference from the control, while red symbols denote a difference from TMZ. Abbreviations: o-CA: Ortho-coumaric acid; U87-MG: Human glioblastoma cell line; TMZ: Temozolomide.

At 24 h, all o-CA derivatives significantly reduced U87-MG cell migration compared to untreated control cells. Notably, Butyl o-CA exhibited the most pronounced inhibitory effect, with an estimated marginal mean wound closure of 45.4%, while control cells achieved 85.0% wound closure (Figure 4; Table S3).

Propyl o-CA treatment (63.2%) produced a wound closure reduction comparable to that observed with TMZ (63.3%). Similarly, Methyl o-CA, Ethyl o-CA, and Isobutyl o-CA treatments resulted in intermediate levels of wound closure (69.0%, 70.0%, and 71.3%, respectively). Dunnett-adjusted post hoc comparisons revealed that Butyl o-CA was the only treatment that induced a significantly greater reduction in wound closure compared to TMZ (Figure 4; Table S3).

### Induction of cell cycle arrest

Cell cycle phase distribution (G0/G1, S, and G2/M) was further examined by reporting estimated marginal probabilities derived from a multinomial regression model. Treatment of U87-MG cells with individual o-CA derivatives revealed distinct effects on cell cycle dynamics depending on the specific compound.

**Figure 5.**
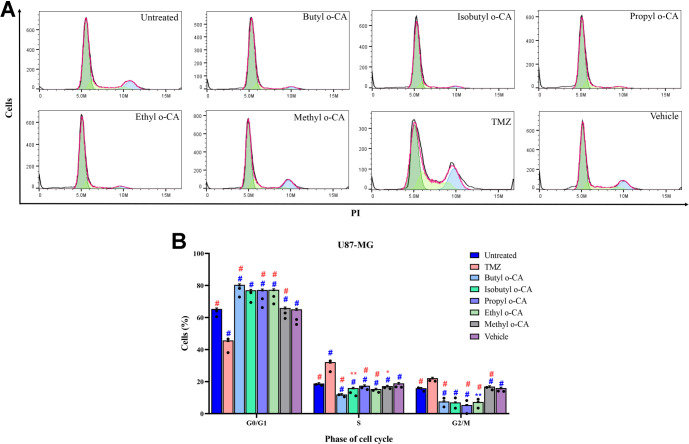
**Effect of o-CA derivatives on cell cycle phases in U87-MG cells**. (A) Representative histograms depict the distribution of cell cycle phases analyzed via PI staining (G0/G1, bright green; S, light green; and G2/M, light blue). (B) Graphical summary illustrating the distribution of U87-MG cells across various cell cycle phases following treatment with o-CA derivatives, TMZ, and vehicle control. Data are presented as estimated marginal probabilities (%) for the G0/G1, S, and G2/M phases, derived from a multinomial regression model with 95% confidence intervals. Dunnett’s adjusted post hoc comparisons were utilized to assess differences relative to the untreated control and TMZ. *P* < 0.05; *P < 0.01; ***P < 0.001; #*P* < 0.0001. Blue symbols indicate statistically significant differences from the control, while red symbols denote differences from TMZ. Abbreviations: o-CA: Ortho-coumaric acid; U87-MG: Human glioblastoma cell line; PI: Propidium iodide; G0/G1: Gap 0/Gap 1 phase; S: Synthesis phase; G2/M: Gap 2/Mitosis phase; TMZ: Temozolomide.

All o-CA compounds induced a significant accumulation of cells in the G0/G1 phase compared to untreated control cells. Among these, Butyl o-CA produced a marked increase in the estimated probability of G0/G1-phase cells (80.4%), followed by Ethyl o-CA (77.33%), Propyl o-CA (77.20%), Isobutyl o-CA (77.02%), and Methyl o-CA (65.93%), indicating a redistribution of cells toward the G0/G1 checkpoint. In contrast, TMZ treatment significantly decreased the estimated probability of G0/G1-phase cells (45.68%) relative to control cells (Figure 5; Table S4).

With respect to the S phase, Butyl o-CA treatment significantly reduced the estimated probability of cells in this phase (11.98%) compared to untreated control cells (18.81%). A similar reduction was observed following treatment with Isobutyl o-CA (15.97%), Ethyl o-CA (15.35%), Propyl o-CA (17.42%), and Methyl o-CA (17.19%). Conversely, TMZ treatment markedly increased the estimated probability of cells in the S phase (32.16%) relative to both untreated and vehicle control cells, as well as all o-CA derivative–treated groups (Figure 5; Table S4).

In the G2/M phase, Propyl o-CA (5.38%), Isobutyl o-CA (7.01%), Ethyl o-CA (7.32%), and Butyl o-CA (7.63%) reduced the G2/M cellular population, while Methyl o-CA (16.88%) showed comparable results to untreated control cells (15.84%), supporting the hypothesis of G0/G1 phase arrest. In contrast, TMZ-treated cells displayed the highest accumulation (22.16%), reinforcing its effect on mitotic arrest (Figure 5; Table S4).

### Expression of the drug resistance marker MGMT

**Figure 6.**
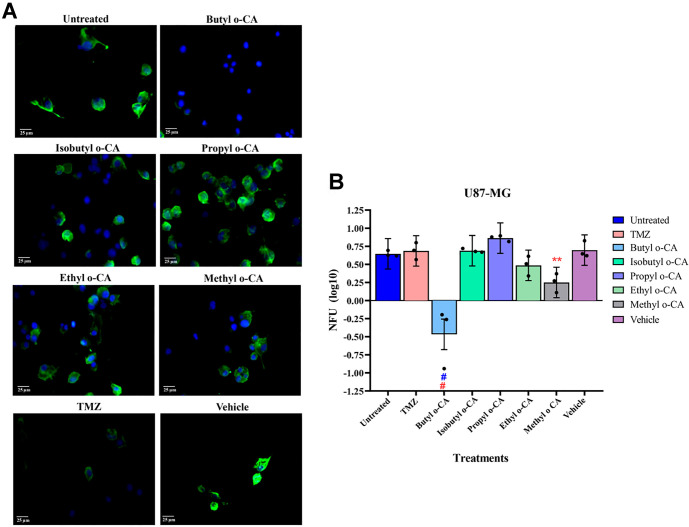
**Effect of o-CA derivatives on the expression of MGMT in U87-MG cells.** (A) Immunofluorescence analysis of MGMT in the cytoplasm and nucleus (green), with nuclei counterstained with DAPI (blue). Scale bar: 25 µm. (B) Quantification of MGMT fluorescence intensity. Each dot represents one biological replicate (mean of 3–5 cells). Data are expressed in NFU, log10-transformed, and presented as mean ± 95% CI. Statistical analysis was conducted using One-way ANOVA followed by Dunnett’s post hoc test compared to control and TMZ; *P* < 0.05; *P < 0.01; ***P < 0.001; #*P* < 0.0001. Blue symbols indicate statistical significance compared to control, while red symbols indicate significance compared to TMZ. Abbreviations: o-CA: Ortho-coumaric acid; MGMT: O6-methylguanine DNA methyltransferase; U87-MG: Human glioblastoma cell line; SLGCs: Stem-like glioblastoma cells; DAPI: 4′,6-diamidino-2-phenylindole; NFU: Normalized fluorescence units; CI: Confidence interval; ANOVA: Analysis of variance; TMZ: Temozolomide.

An immunofluorescence assay was conducted to determine whether o-CA derivatives modulate MGMT expression, a protein involved in DNA repair, in U87-MG cells. Basal MGMT expression was detected in both the untreated control (mean log_1__0_ NFU = 0.645) and the vehicle group (0.697). The most pronounced reduction in MGMT expression was observed in cells treated with Butyl o-CA (-0.467), reaching levels significantly lower than both control and TMZ-treated cells (0.686). A moderate decrease in MGMT was also detected following treatment with Methyl o-CA (0.250) and Ethyl o-CA (0.486). In contrast, Isobutyl o-CA (0.689) and Propyl o-CA (0.863) exhibited MGMT expressions comparable to control and TMZ levels. Only Butyl o-CA and Methyl o-CA showed significantly decreased MGMT expression compared to TMZ (Figure 6; Table S5).

### Autophagy induction via LC3-B expression

**Figure 7.**
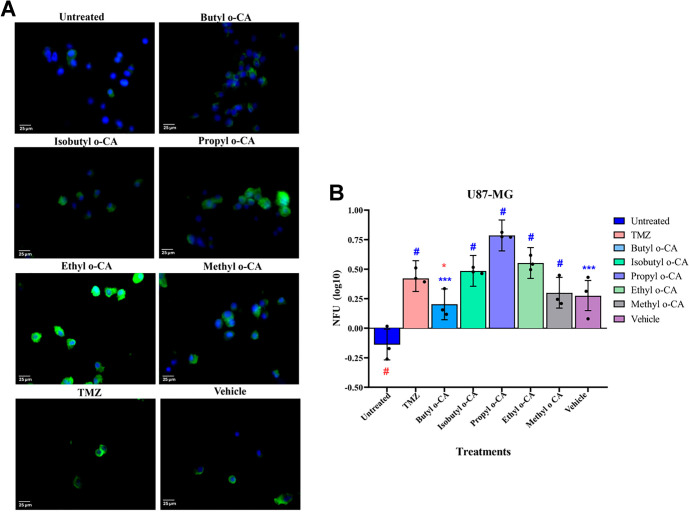
**Effect of o-CA derivatives on the expression of the autophagy marker LC3-B in U87-MG cells.** (A) Immunofluorescence analysis showing LC3-B (green) with nuclei counterstained using DAPI (blue). Scale bar: 25 µm. (B) Quantification of LC3-B fluorescence intensity. Each dot represents one biological replicate (mean of 3-5 cells). Data are presented as NFU, log10-transformed, and expressed as mean ± 95% CI. Statistical analysis was conducted using one-way ANOVA followed by Dunnett’s post hoc test compared to control and TMZ; *P* < 0.05; *P < 0.01; ***P < 0.001; #*P* < 0.0001. Blue symbols indicate statistical significance versus control, while red symbols indicate significance versus TMZ. Scale bar: 25 µm. Abbreviations: o-CA: Ortho-coumaric acid; LC3-B: Microtubule-associated protein 1 light-chain 3B; U87-MG: Human glioblastoma cell line; SLGCs: Stem-like glioblastoma cells; DAPI: 4′,6-diamidino-2-phenylindole; NFU: Normalized fluorescence units; ANOVA: Analysis of variance; TMZ: Temozolomide; CI: Confidence interval.

To assess whether o-CA derivatives promote autophagy, LC3-B expression, a key protein in autophagosome formation and maturation, was evaluated. Basal LC3-B expression was minimally detected in both the untreated control (mean log_1__0_ NFU ═ –0.139) and the vehicle group (0.274). TMZ treatment induced a moderate increase in LC3-B levels (0.482), consistent with the known activation of stress-related autophagy pathways. Among the o-CA derivatives, Propyl o-CA exhibited the highest LC3-B expression (0.786), followed by Ethyl o-CA (0.552), Isobutyl o-CA (0.485), and Methyl o-CA (0.300). In contrast, Butyl o-CA demonstrated the lowest expression among the derivatives (0.203), suggesting minimal induction of autophagy. These results indicate that specific o-CA derivatives, particularly Propyl o-CA, may increase LC3-B levels in a manner consistent with altered autophagy-related activity in U87-MG cells (Figure 7; Table S6).

### Expression of differentiation and stem markers in SLGCs

To evaluate the effects of o-CA derivatives on stemness and differentiation, the expression of CD133, Nestin, GFAP, and NeuN was assessed via immunofluorescence. CD133 and Nestin served as markers of SLGCs, while GFAP and NeuN were utilized as markers of glial and neuronal differentiation, respectively.

**Figure 8.**
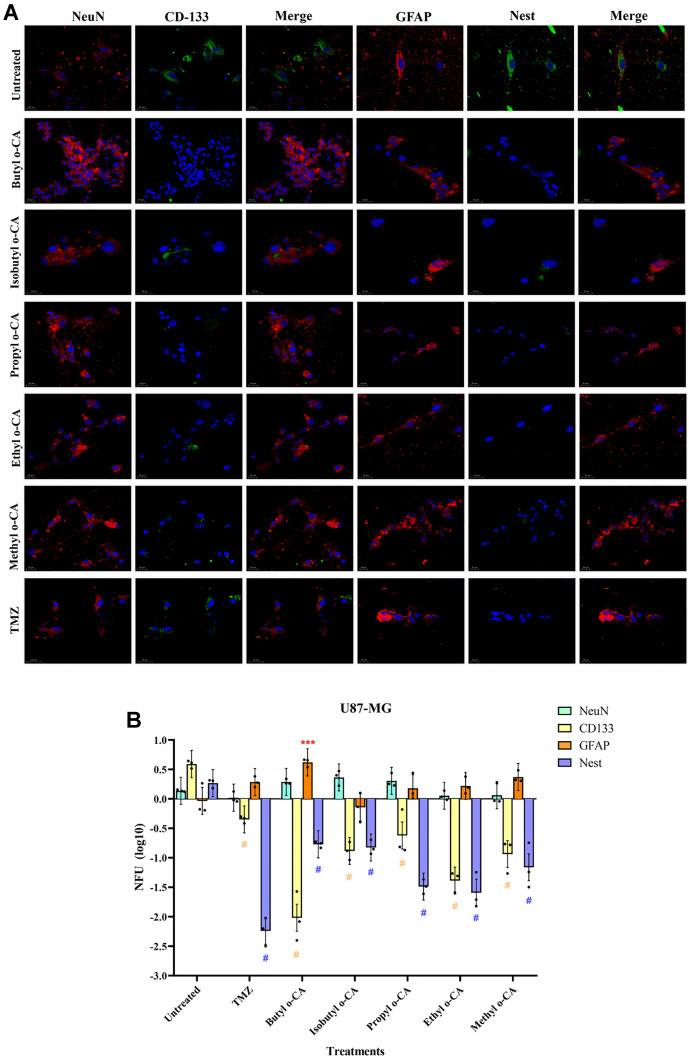
**Effect of o-CA derivatives on the expression of differentiation and stemness markers in U87-MG cells.** (A) Immunofluorescence analysis was conducted to assess differentiation markers GFAP and NeuN (red) alongside stemness markers CD133 and Nestin (green). Nuclei were counterstained with DAPI (blue) in U87-MG cells treated with o-CA derivatives. Scale bar: 20 µm. (B) Quantification of fluorescence intensity for NeuN, CD133, GFAP, and Nestin is presented. Each dot represents one biological replicate (mean of 3–5 cells). Data are expressed in NFU, log10-transformed, and presented as mean ± 95% CI. Statistical analysis was performed using Two-way ANOVA followed by Dunnett’s post hoc test compared to control; *P* < 0.05; *P < 0.01; ***P < 0.001; #*P* < 0.0001.Colored symbols indicate statistical significance: Green for NeuN, yellow for CD133, orange for GFAP, and purple for Nestin. Abbreviations: O-CA: Ortho-coumaric acid; U87-MG: Human glioblastoma cell line; SLGCs: Stem-like glioblastoma cells; GFAP: Glial fibrillary acidic protein; NeuN: Neuronal nuclei; DAPI: 4′,6-diamidino-2-phenylindole; NFU: Normalized fluorescence units; CI: Confidence interval; ANOVA: Analysis of variance.

NeuN expression in untreated control U87-MG cells was low (0.136) compared to treatment with o-CA derivatives, which resulted in differential modulation of NeuN levels. A marked increase was observed in Isobutyl o-CA–treated cells (0.363), followed by Propyl o-CA (0.306) and Butyl o-CA (0.287). Conversely, NeuN expression in Methyl o-CA (0.063) and Ethyl o-CA (0.053) treated cells remained comparable to control levels. TMZ-treated cells exhibited lower NeuN expression (0.019) relative to control and o-CA derivatives (Figure 8; Table S7).

CD133 expression was highest in control cells (0.590) and remained detectable following TMZ treatment (--0.348). In contrast, all o-CA derivatives significantly reduced CD133 expression, with the most pronounced decrease observed with Butyl o-CA (--2.018), followed by Ethyl o-CA (-1.387), Methyl o-CA (--0.938), Isobutyl o-CA (-0.883), and Propyl o-CA (--0.621).

GFAP, a marker involved in astrocytic/glial differentiation, exhibited relatively low expression in control cells (--0.035) and was differentially affected by treatments. A significant increase was detected in Butyl o-CA–treated cells (0.619) compared to untreated cells, followed by cells treated with Methyl o-CA (0.371) and Ethyl o-CA (0.219). Propyl o-CA induced a moderate increase in GFAP expression (0.180), while Isobutyl o-CA treatment resulted in reduced GFAP levels (-0.143). GFAP expression in TMZ-treated cells showed levels similar (0.286) to those of Ethyl o-CA.

Finally, Nestin expression was significantly reduced in all o-CA–treated groups compared to untreated control cells (0.267). The lowest Nestin levels were observed in Ethyl o-CA (--1.592) and Propyl o-CA (-1.488) treated cells, followed by Methyl o-CA (--1.161), Isobutyl o-CA (--0.826), and Butyl o-CA (--0.770). TMZ treatment exhibited the most pronounced reduction in Nestin expression overall (-2.241) compared to o-CA derivatives.

Collectively, these findings indicate that o-CA derivatives modulate stemness and differentiation-associated markers in a compound-dependent manner. While all derivatives consistently reduced stem-like markers, their effects on neuronal and glial differentiation markers varied among compounds, suggesting selective rather than uniform differentiation-related responses in U87-MG cells (Figure 8).

## Discussion

Phenolic compounds found in foods, such as caffeic, ferulic, sinapinic, and para-coumaric acid (p-CA), have been associated with anticancer properties, including the induction of apoptosis and a reduction in cell migration and proliferation [26, 27]. However, the study of individual phenolic compounds is often limited by their low abundance and presence in complex mixtures within natural extracts. To overcome these limitations, chemical and enzymatic approaches have been developed to synthesize hydroxycinnamic acid derivatives, such as o-CA esters, allowing for the evaluation of their biological activity under controlled conditions [28–30].

The potency pattern of biological activity among o-CA derivatives (Butyl > Isobutyl > shorter chains) may be explained by their structure–activity relationships (SAR). Longer alkyl chains, as seen in Butyl o-CA, may confer favorable lipophilicity (Log *P* ═ 2.78), enhancing membrane interactions and intracellular availability. Conversely, alkyl chain length and branching (steric hindrance), as in Isobutyl o-CA, can modulate the rate of esterase-mediated hydrolysis, increasing molecular stability [31]. It is well established that ester derivatives may function as prodrugs, with their intracellular availability influenced by esterase-mediated hydrolysis, consistent with SAR trends reported for hydroxycoumarins and their derivatives [13, 32, 33].

Previous studies have reported anticancer effects of o-CA and related compounds in various solid tumor models, including NCI-H460 lung cancer cells, and have demonstrated cytotoxic effects in tumor necrosis factor-related apoptosis-inducing ligand (TRAIL)-resistant prostate cancer LNCaP and DU-145 cell lines [34, 35]. Additionally, studies in breast cancer MCF-7 cells have shown that o-CA increases the expression of pro-apoptotic proteins and induces cell cycle arrest [36]. These findings provide a plausible mechanistic framework supporting the antitumor effects observed in our results with o-CA and U87-MG cells.

The U87-MG glioblastoma cell line is widely utilized as a model for studying tumor heterogeneity due to its heterogeneous subpopulations, which exhibit both stem-like and differentiated phenotypes. Prior research has shown that U87-MG cells contain CD133^+^ cancer stem-like subpopulations that differ from their differentiated counterparts in tumorigenic properties [15, 37]. Consequently, U87-MG serves as an appropriate model for assessing clonogenicity, tumor spheroid formation, cell cycle regulation, and the modulation of molecular markers related to stemness, differentiation, DNA repair, and autophagy.

Building upon this foundation, the present study extends these observations by demonstrating a consistent impairment of stem-like glioblastoma cell properties in o-CA derivatives. Our results collectively indicate that o-CA derivatives impede self-renewal capacity by reducing tumor sphere and colony formation, inducing cell cycle arrest, decreasing migratory potential, and modulating molecular markers associated with drug resistance, autophagy, and differentiation. Notably, these effects were compound-dependent, with long-chain derivatives such as Butyl o-CA and Isobutyl o-CA exhibiting the most significant and consistent effects compared to shorter-chain derivatives (Propyl, Ethyl, and Methyl o-CA).

Spheroid-forming capacity was evaluated using a serum-derived tumor spheroid model, a well-established three-dimensional system for glioblastoma research (Günther et al., 2012; Yu et al., 2012). This tumor spheroid formation assay demonstrated that o-CA derivatives impaired spheroid formation and induced pronounced morphological alterations, including reduced size and irregular edges, which are consistent with disrupted cell–cell interactions and stem-like properties. These effects were most pronounced for Butyl o-CA and Isobutyl o-CA, which significantly reduced both spheroid formation efficiency and clonogenic capacity in U87-MG cells.

Butyl o-CA and Isobutyl o-CA exhibited the strongest inhibitory effects on spheroid formation. Clonogenic assays reinforced these findings, revealing that Isobutyl o-CA and Butyl o-CA markedly reduced colony formation compared with other o-CA derivatives. Importantly, these inhibitory effects surpassed those observed with TMZ, suggesting that selected o-CA derivatives may operate through mechanisms that are either independent of or complementary to TMZ-mediated cytotoxicity.

Spheroid formation and clonogenic survival reflect the long-term proliferative and self-renewal capacities of individual tumor cells [38, 39]. The concomitant inhibition of both parameters observed here supports a robust impairment of glioblastoma stem-like properties. However, we acknowledge that the reduced spheroid and colony formation following o-CA treatment may partially reflect decreased cell survival, indicating a combination of cytotoxic/cytostatic and stemness-related effects that cannot be fully dissociated within the current experimental design.

In parallel with the inhibition of self-renewal, o-CA derivatives significantly reduced the migratory capacity of U87-MG cells, with Butyl o-CA exhibiting the most pronounced anti-migratory effect, surpassing that of TMZ. These results imply that Butyl o-CA has a significant anti-migratory effect on glioblastoma cells. It is important to note that scratch wound closure assays performed in the presence of serum reflect the combined contributions of cell migration, proliferation, and survival. Cell migration is a key determinant of glioblastoma aggressiveness and is closely associated with stem-like phenotypes (CD133+) and therapeutic resistance [40]. Previous reports indicate that phenolic compounds, including flavonoids, can inhibit migration and invasion in glioblastoma [41]. Additionally, ortho-coumaric molecules have been shown to reduce cell migration capacity in various cancer types, including lung and breast cancer [42, 43]. Thus, o-CA may possess similar therapeutic potential against glioblastoma.

Consistent with their functional effects, o-CA derivatives induced cell cycle arrest at the G0/G1 phase, accompanied by a concomitant reduction in the S and G2/M phases. This pattern contrasts with TMZ treatment, which promotes S-phase accumulation and G2/M arrest, consistent with its canonical DNA-damaging mechanism of action.

Previous studies have demonstrated that structurally related phenolic compounds, such as p-coumaric acid, can modulate cell cycle regulators and mitogen-activated protein kinase (MAPK) signaling pathways, leading to cell cycle arrest and reduced proliferative capacity across various tumor models, including melanoma [44, 45] and glioblastoma U87-MG cells [46]. The G0/G1 arrest induced by o-CA derivatives suggests a predominantly cytostatic effect, potentially limiting proliferative expansion and long-term self-renewal, thereby contributing to the observed reductions in clonogenic capacity and tumor sphere-forming efficiency. To further elucidate the molecular mechanisms underlying these functional effects, we next examined the expression of MGMT, a key marker associated with DNA repair and resistance in glioblastoma cells.

MGMT is a critical DNA repair enzyme and a major determinant of resistance to alkylating agents in glioblastoma, serving as a functional marker associated with GBM tumor cells. Previous research has indicated that bioactive compounds, such as curcumin, can downregulate MGMT and other anti-apoptotic and DNA repair proteins, including Bcl-2 and Bcl-xL, thereby sensitizing tumor cells to radiotherapy and chemotherapy [47–49].

A particularly relevant finding of the present study is the compound-specific modulation of MGMT expression by o-CA derivatives. Among the tested compounds, Butyl o-CA induced a marked downregulation of MGMT expression, reaching levels significantly lower than those observed in control and TMZ-treated cells, while TMZ did not suppress MGMT expression, consistent with the well-documented feedback induction of MGMT in response to alkylating DNA damage and a key mechanism underlying TMZ resistance in glioblastoma [50]. Conversely, Propyl o-CA exhibited an upward trend in MGMT expression, further supporting a compound-specific regulatory effect on this DNA repair pathway. The ability of Butyl o-CA to suppress MGMT expression suggests a potential mechanism through which this compound may preferentially sensitize SLGCs, which rely on efficient DNA repair for survival and therapeutic resistance, aligning with previous reports that phenolic compounds can modulate DNA repair and tumor suppressor pathways, ultimately leading to impaired proliferation and increased susceptibility to cell death [51].

Analysis of LC3-B expression revealed that o-CA derivatives exert compound-specific effects on autophagy-related activity in U87-MG cells. Propyl o-CA induced the highest increase in LC3-B levels, whereas Butyl o-CA demonstrated minimal LC3-B induction despite its strong inhibitory effects on self-renewal and migration. This lack of correlation suggests that autophagy induction is not the primary mechanism underlying the antitumor activity of o-CA derivatives but rather reflects a differential cellular stress response. Additional markers of autophagic flux would be necessary to determine the functional relevance of these observations.

Autophagy is a context-dependent process in cancer, acting either as a cytoprotective mechanism or as a form of programmed cell death, particularly in apoptosis-resistant tumors such as glioblastoma [52]. Prior studies have shown that polyphenolic compounds can promote autophagy [53], including honokiol, which can induce apoptosis and autophagy-associated markers such as Beclin-1 and microtubule-associated protein 1A/1B-light chain 3-II (LC3-II) in glioblastoma cells, often in parallel with apoptosis and reduced cell viability, underscoring the complexity of autophagy modulation in this tumor type [54]. Accordingly, [46] reported the induction of autophagy-related protein p62 by p-coumaric acid. Thus, the increased LC3-B expression observed following Propyl o-CA treatment may reflect a similar stress-adaptive autophagic response.

The o-CA derivatives consistently reduced the expression of canonical stem cell markers CD133 and Nestin, closely associated with self-renewal capacity, tumor initiation, and therapeutic resistance in glioblastoma [55]. This molecular profile aligns with the marked reduction in tumor sphere formation and clonogenic capacity observed following treatment, indicating a coordinated impairment of stem-like properties rather than a solely cytotoxic effect. Similar attenuation of stem-like states has been reported for other phenolic compounds in glioblastoma models [56].

Recent evidence demonstrates that corosolic acid, a plant-derived triterpenoid, markedly suppresses glioblastoma cancer stem-like cell properties, including sphere formation, clonogenicity, migration, and invasion, concomitant with downregulation of core stemness regulators such as CD133, octamer-binding transcription factor 4 (OCT4), and Nestin [57]. Consistent with these observations, our data indicate that o-CA derivatives induce heterogeneous modulation of differentiation-associated markers, with partial increases in NeuN and GFAP expression in selected treatments. Evidence from non-neoplastic stem cell systems further suggests that coumaric acids can influence differentiation-related regulatory pathways, including peroxisome proliferator-activated receptor gamma (PPARγ) signaling [58], which has been proposed as a relevant therapeutic target in glioblastoma [59]. Collectively, these findings support the notion that phenolic and related natural compounds modulate glioblastoma cellular plasticity and may promote a shift toward a less stem-like and less aggressive phenotype.

Certain limitations should be acknowledged. All experiments were conducted using a single established GBM cell line (U87-MG), which only partially represents the genetic and phenotypic heterogeneity observed in human brain tumors. Furthermore, although key functional parameters, including cell viability, migration, autophagy, and stemness/differentiation markers, were analyzed, no patient-derived primary cultures or physiologically relevant 3D models were included. Future studies should validate these findings in patient-derived spheroids or organoid models that better recapitulate GBM heterogeneity. Additionally, transcriptomic, proteomic, and metabolomic profiling could uncover molecular targets and clarify the pathways modulated by o-CA derivatives, ultimately strengthening their potential as therapeutic candidates.

## Conclusion

In summary, this study provides *in vitro* evidence that o-CA derivatives, particularly Butyl and Isobutyl o-CA, impair key tumorigenic properties of glioblastoma stem-like cells, including sphere formation, clonogenicity, migration, and cell cycle progression, suggesting reduced self-renewal and proliferative potential. These functional effects are supported by molecular changes such as MGMT downregulation, modulation of the autophagy-related marker LC3-B, and a coordinated decrease in stemness markers CD133 and Nestin, alongside increased expression of neural and glial differentiation markers NeuN and GFAP, indicating a shift toward a less aggressive cellular phenotype. Collectively, these findings position o-CA derivatives as promising modulators of glioblastoma behavior and provide a rationale for further mechanistic and combinatorial studies, rather than definitive evidence of enhanced therapeutic efficacy.

## Supplemental data

Supplemental data are available at the following link: https://www.bjbms.org/ojs/index.php/bjbms/article/view/13514/4212

## Data Availability

The data that supports the findings of this study can be requested from the corresponding author.
